# Proportion trends, cancer stage, and survival of patients with cancer diagnosed through emergency and nonemergency departments: a nationwide cohort study

**DOI:** 10.3389/fonc.2024.1399326

**Published:** 2024-08-26

**Authors:** Ying-Chao Lin, Wei-Yin Kuo, Pei-Tseng Kung, Wen-Chen Tsai

**Affiliations:** ^1^ Graduate Institute of Public Health, China Medical University, Taichung, Taiwan; ^2^ School of Medicine, Tzu Chi University, Hualien, Taiwan; ^3^ Department of Neurological Institute, Taichung Tzu Chi Hospital, Buddhist Tzu Chi Medical Foundation, Taichung, Taiwan; ^4^ Department of Health Services Administration, China Medical University, Taichung, Taiwan; ^5^ Department of Healthcare Administration, Asia University, Taichung, Taiwan; ^6^ Department of Medical Research, China Medical University Hospital, China Medical University, Taichung, Taiwan

**Keywords:** breast cancer, lung cancer, oral cancer, colorectal cancer, prostate cancer, emergency department, survival, cancer stage

## Abstract

**Introduction:**

To reduce mortality, the Taiwan government has vigorously promoted free cancer screening and preventive health screening services. Cancers are usually advanced by the time they are discovered in the emergency department. Through this study, we aimed to understand the characteristics of cancer patients diagnosed through the emergency department and thus identify high-risk populations by comparing cancer staging and survival rates in patients diagnosed in the emergency department and those diagnosed in the non-emergency department.

**Methods:**

The retrospective study enrolled a total of 389,043 patients over the age of 20 who were newly diagnosed with one of the five major cancers (including lung cancer, colorectal cancer, breast cancer, prostate cancer, and oral cancer) between 2008 and 2017 and analyzed their diagnostic pathway, cancer stage at diagnosis, and survival time.

**Results:**

Of the study participants, 59,423 patients (about 15.3%) were diagnosed with cancer through the emergency department. We found that a sizable proportion of older people and patients with low education and low incomes were diagnosed through emergency department visits, and those with a health condition comorbidity severity of 3 had the highest proportion diagnosed by the emergency department, advanced stages at diagnosis, and risk of death. These can be classified as high-risk groups. In addition, 76.4% of patients diagnosed in the emergency department had advanced cancer, and the risk of death was 1.46 times higher than that of patients diagnosed in the non-emergency department. Although cancer screening is available, it does not reduce the proportion of patients with advanced cancer who are diagnosed through or at the time of diagnosis in the emergency department.

**Conclusions:**

The present study found that the government’s cancer screening did not affect the proportion or number of cancers diagnosed through emergency department visits. Therefore, the government should focus on more cancer screening, health education in high-risk groups, and strengthening the link between emergency and oncology departments to reduce the risk of death for patients diagnosed through emergency department visits.

## Introduction

Cancer has always been a major global health issue. According to the 2021 cancer registry report by the Health Promotion Administration of Taiwan, 121,762 people were newly diagnosed with cancer in 2021, with a crude incidence rate of 520.9 per 100,000 people and an age-standardized incidence of 306.5. The six most common cancers were lung, colorectal, breast, liver, oral, and prostate cancers (1). The 2021 cancer registry report of Taiwan disclosed that the main stages of breast, colorectal, and prostate cancers at diagnosis were stages 0 to 2, while those of lung and oral cancers were stages 3 to 4 (1). Cancer ranks first among the top 10 causes of death in Taiwan. From 2017 to 2021, the 5-year relative survival for the top 10 cancers in Taiwan was 62.1%, including 90.2% for breast cancer, 87.1% for prostate cancer, 64.1% for colorectal cancer, 57.0% for oral cancer, 40.1% for lung cancer, and 36.4% for liver cancer (1). The survival rates of liver cancer and lung cancer were low.

Cancer staging is one of the main factors affecting the survival rate of patients with cancer: the survival rate decreases as the cancer stage increases (2, 3). Early diagnosis and treatment of cancer may significantly improve survival rates (4, 5); therefore, surveilling high-risk groups is important for early cancer diagnosis (6). The Taiwan’s government aimed to reduce cancer mortality and prevent cancer from harming people’s health through early detection and treatment. To this end, the government planned free screening services for the four major cancers (breast, cervical, colorectal and oral cancers). Statistics show that patients who undergo cancer screening have lower cancer staging when diagnosed compared to those who do not undergo screening (7–9). Patients who undergo more screening tests have a higher chance of being diagnosed with early-stage cancer (8).

In addition to being detected through screening, cancer is mostly identified when patients seek medical treatment for symptoms. When patients visit an emergency department with critical symptoms, the severity of their disease can also vary. Research statistics from some countries indicate that most patients diagnosed with cancer through outpatient clinics have early-stage cancer, while most patients diagnosed through the emergency department have advanced cancer with relatively severe symptoms (10–12). Studies have compared differences between patients with cancer diagnosed in outpatient clinics and those diagnosed through emergency departments: the cancer stage of patients diagnosed through emergency departments was found to be more advanced than that of patients diagnosed in outpatient clinics. Moreover, the condition of patients diagnosed with cancer through emergency departments was often life-threatening, and their prognosis was worse than that of patients diagnosed through outpatient clinics (13).

The routes to diagnosis are through outpatient clinics and emergency departments. A study conducted in the United Kingdom used data from the English Cancer Patient Experience Survey in 2010 to analyze 4,647 emergency cases with 18 different cancers. The researchers determined that 1,349 (29%) patients diagnosed with cancer had no cancer-related outpatient medical records (11). The prognosis of patients diagnosed with cancer through the emergency department was worse than that of patients diagnosed through the outpatient clinic. For example, the cancer stage of colorectal, lung, breast, and prostate cancers in patients diagnosed through the emergency department is more advanced than that of the same cancers diagnosed through the outpatient clinic (10). Analysis of cancer diagnosis cases through the emergency department showed that 30% of patients were diagnosed with stage 4 cancer; of those patients diagnosed with cancer through the emergency department, 33%, 32%, 59%, and 39% of patients were diagnosed with stage 4 breast, colorectal, lung, and prostate cancers, respectively (13).

Breast, colorectal, lung, prostate, and oral cancers are five of the top six cancers in Taiwan. To date, in Taiwan, no studies have explored the difference in cancer stage and survival between patients diagnosed in outpatient clinics and those diagnosed in the emergency department. Therefore, in the current study, the probability and risks of diagnosis of the five major cancers in Taiwan through outpatient clinics and emergency departments were analyzed. The differences in cancer stage and related factors for mortality risks were also compared between routes to diagnosis, and the differences in routes to diagnosis and cancer stage at diagnosis were compared between the presence and absence of a free cancer screening policy. These findings may serve as a reference for formulating future cancer prevention strategies.

## Methods

### Data sources

This study adopts a retrospective cohort study design. The data for this study was obtained from the Taiwan cancer registry files (managed by the Health Promotion Administration) for the period 2008 to 2017, which were used to recruit research participants, their cancer stage at diagnosis, and date of diagnosis. We also associate this data with the National Health Insurance Research Database (NHIRD), the cause of death file of the Ministry of Health and Welfare, and household registration data from 2006 to 2018 to obtain the route to diagnosis, personal characteristics, economic status, environmental factors, health status, cancer treatment, characteristics of the main medical institutions, and mortality of the participants. Diagnose cancer according to the third edition of the International Classification of Oncology Diseases (ICD-O-3), which identifies cancer categories according to the primary site, histology, behavior code, and classification. All cancer diagnosis dates were based on the cancer registry file records. The NHIRD consists of all medical records, including outpatients, emergency department (ED) visits, and inpatient records. Since the cancer registry file does not provide all cancer patient medical records, we need to link the cancer registry file to the National Health Insurance Research Database, which provides all medical records for all patients in Taiwan. The outpatient and ED medical records were obtained from “the Outpatient Care Files,” and the inpatient medical records were obtained from “the Inpatient Care Files” in the NHIRD.

### Study population

**Figure 1:**
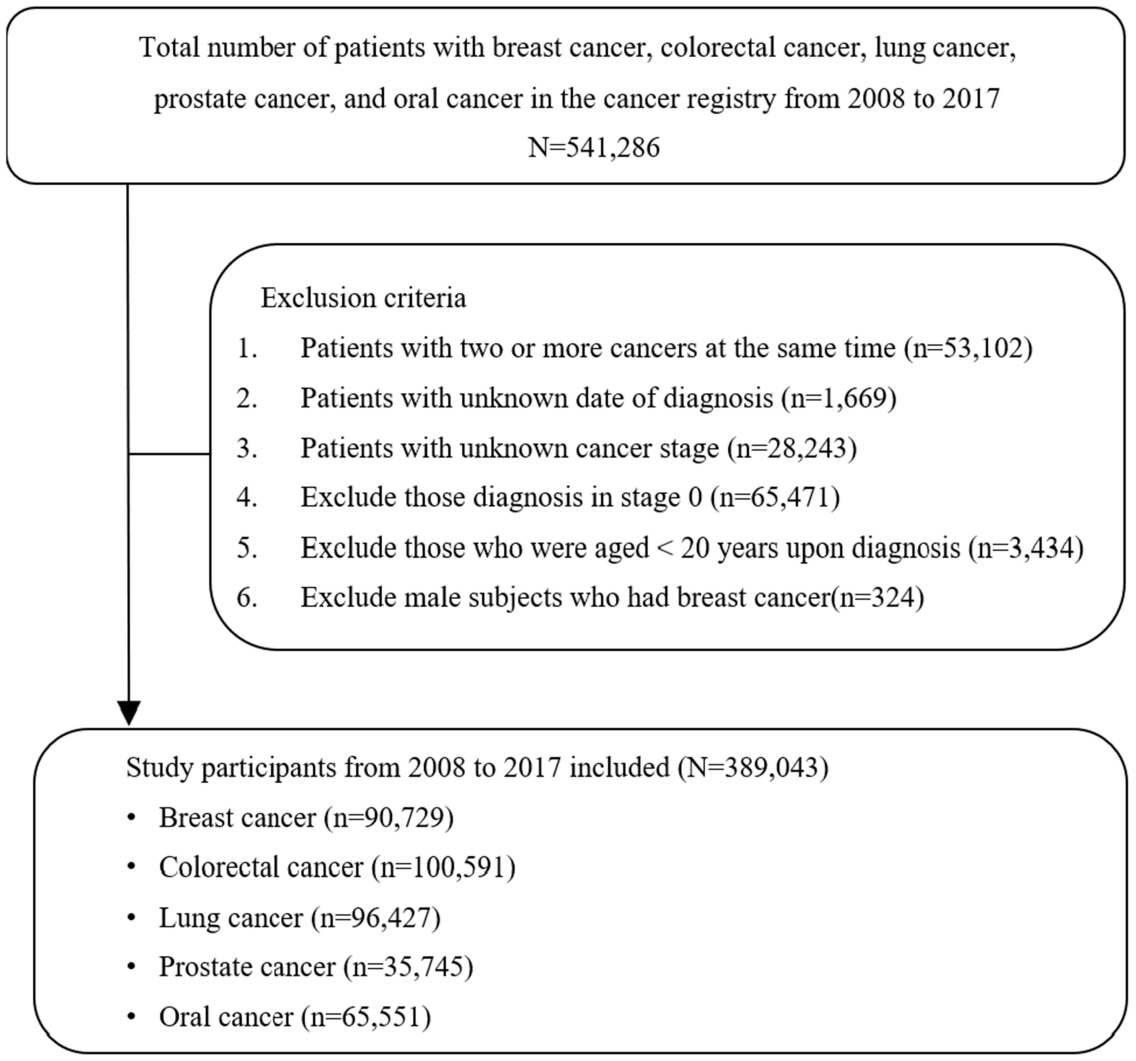
Detailed schematic of the enrollment process for study participants.

This study focused on patients aged ≥20 years old who were newly diagnosed with one of the five major cancers between 2008 and 2017, including female breast cancer (ICD-O-3 is C50), colorectal cancer (ICD-O-3 is C18-C21), lung cancer (ICD-O-3 is C33-C34), prostate cancer (ICD-O-3 is C61), and oral cancer (ICD-O-3 is C00-C14). In total, 541,286 patients were diagnosed with the five cancers during the study period. After excluding patients who had two or more cancers simultaneously, unknown cancer stage at diagnosis, stage 0, and incomplete data for research variables, a total of 389,043 participants were included in the analysis. Among them, 90,729 patients had breast cancer, 100,591 had colorectal cancer, 96,427 had lung cancer, 35,745 had prostate cancer, and 65,551 had oral cancer. The detailed research participant enrollment process is shown in **Figure 1**. The cancer-relevant diagnosis codes were listed in the **Supplementary Appendix**.

### Study variables

**Figure 2:**
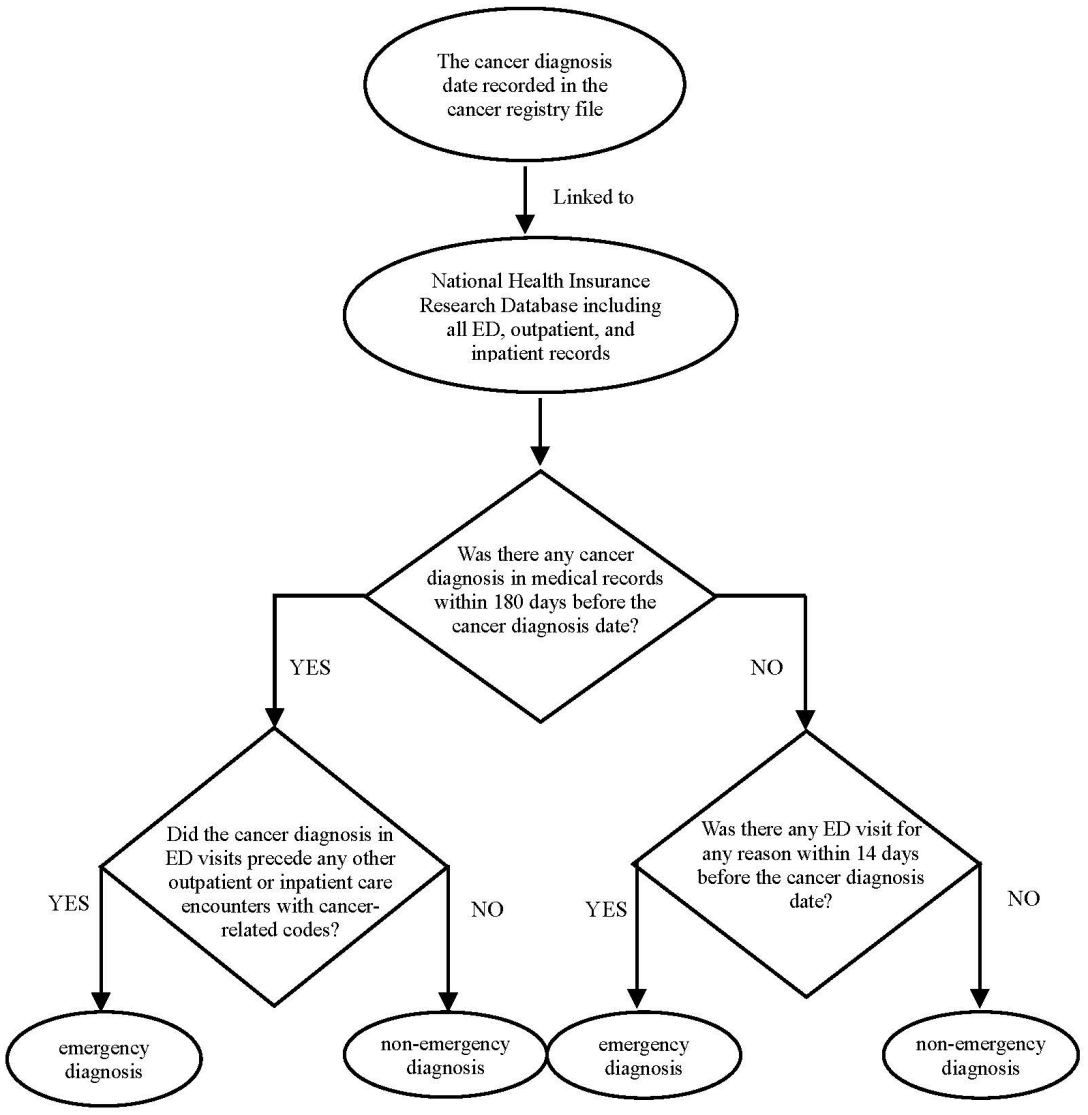
Flow chart of the cancer diagnosis route.

Route to diagnosis, stage at diagnosis, and survival were used as dependent variables. We obtained information on cancer diagnosis and date of diagnosis (and stage at diagnosis) from the Taiwan cancer registry, and in subsequent steps, we have classified each cancer case either as emergency department-diagnosed or non-emergency department-diagnosed. We have chiefly used two criteria, as follows:

First, we have examined the linked National Health Insurance Research Database (NHIRD) records of each cancer patient up to 180 days pre-diagnosis, searching for healthcare encounters with cancer-related diagnostic codes. Among cancer patients with a cancer-related diagnostic code in their NHIRD record up to 180 days pre-diagnosis, if the chronologically first occurrence of such code(s) related to an emergency department encounter (which preceded any other outpatient or elective inpatient care encounters with cancer-related codes), we classified such a case as an emergency department-diagnosed. Second, among cancer cases who did not have an NHIRD cancer-relevant code in the 180-day pre-diagnosis, we have additionally examined NHIRD for the occurrence of an emergency department encounter up to -14 days from the cancer registry diagnosis date, and if there has been such an encounter, the patient was classified as emergency department diagnosed. Notably, unlike our first criterion, this second criterion was not restricted to cancer-relevant diagnosis codes, i.e., the mere fact of an emergency department encounter up to -14 days from a cancer diagnosis sufficed for the second criterion to be met (the flow chart of the cancer diagnosis route shown in **Figure 2**).

The tumor, node, and metastasis cancer staging methods of the American Joint Committee on Cancer were applied to the data in the cancer registry to confirm cancer stages of 1, 2, 3, or 4 among the five cancers. The participants were then divided into two groups: one comprising patients with early-stage (stages 1 and 2) disease and the other comprising patients with advanced stage (stages 3 and 4) disease. For survival status, the cause of death data was connected to track whether a participant died after diagnosis. Cause of death was tracked until the end of 2018. Death was defined as an event. Conversely, survival at the end of 2018 or participants withdrawn from the National Health Insurance was defined as censored data.

Other variables included the type of cancer (female breast, colorectal, lung, prostate, and oral), as well as personal characteristics, including sex, age at diagnosis, education level, marital status, monthly salary (≤NT$17,280, NT$17,281–22,800, NT$22,801–28,800, NT$28,801–36,300, NT$36,301–45,800, NT$45,801–57,800, NT$57,801–72,800, or ≥NT$72,801), degree of urbanization of the place of residence (divided into seven grades, with grade 1 being areas with the highest degree of urbanization and grade 7 being areas with the lowest degree of urbanization (14), type of main medical institution attended (medical center, regional hospital, district hospital, or clinic), institutional ownership type (public hospital or nonpublic hospital), and year of diagnosis. Regarding health status, based on the Charlson Comorbidity Index modified by Deyo et al., the primary and secondary diagnosis codes of the patients as per ICD-9-CM were converted into numerical weighted scores to represent the severity of comorbidities. These scores were divided into 0, 1, 2, and ≥3 points, with a higher score indicating more a serious comorbidity (15).

### Statistical analysis

This study used descriptive and inferential statistical analyses. Descriptive statistics such as numbers and percentages were used to demonstrate the annual proportions and trends of the patients with the five major cancers diagnosed through outpatient clinics or emergency departments. The chi squared test was performed to examine differences in cancer types, personal characteristics, economic status, environmental factors, health status, type of main medical institution, and year of diagnosis between patients diagnosed with cancer through the two routes. Differences in diagnostic pathway and distribution of related variables were explored among the patients in the cohort who were diagnosed at early and advanced stages. The log-rank test was then performed to examine the differences in survival among patients with the five major cancers by route to diagnosis, personal characteristics, economic status, environmental factor, cancer stage, health status, cancer treatment, and type of main medical institution, respectively.

Inferential statistical analyses included the use of logistic regression to compare the risk of diagnosis through the emergency department for patients with the five major cancers. Moreover, logistic regression analysis was performed to explore the risk of diagnosis at advanced stages among patients with the five cancers. The Cox proportional hazard model was used to explore the differences in mortality risk between patients with the five cancers who were diagnosed through outpatient and emergency departments. This study was approved by the research ethics committee of China Medical University and Hospital (Institutional Review Board No. CRREC-109-156).

## Results

**Figure 3:**
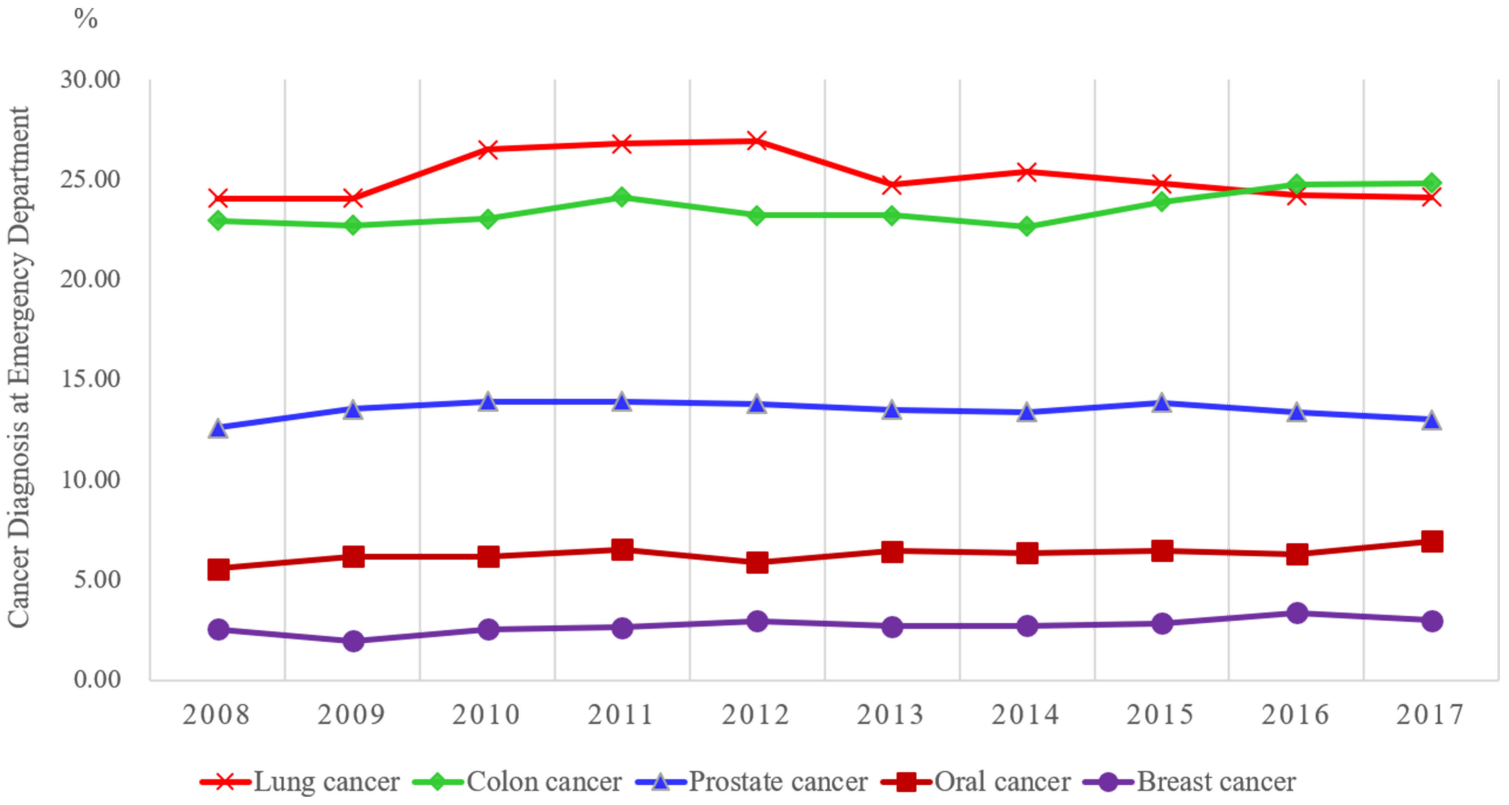
Trend in the proportion of cancer cases diagnosed through the emergency department by year.

The trend in the proportion of patients with cancer diagnosed through the emergency department was first analyzed. A total of 389,043 patients who were newly diagnosed with one of the five major cancers (including breast, colorectal, lung, prostate, and oral cancers) from 2008 to 2017 were enrolled. Among the newly diagnosed patients, the highest proportion of diagnoses through the emergency department was observed for lung cancer (25.1%), followed by colorectal cancer (23.6%). The lowest proportion of diagnoses was observed for breast cancer (2.8%) (**Table 1**). The highest proportion of patients newly diagnosed with stage 4 cancer through the emergency department was observed for colorectal cancer (32.2%), followed by lung cancer (31.2%); the lowest was observed for breast cancer. Of note, is that the proportion of cancer diagnoses through emergency department visits was stable from 2008 to 2017. However, a slight upward trend for breast and colorectal cancer was observed (**Figure 3**).

**Table 1:** The number and staging of cancer diagnoses made through emergency and non-emergency departments from 2008 to 2017.

	Breast cancer					Colon cancer					Lung cancer					Prostate cancer					Oral cancer				
	OPD		ED		P value	OPD		ED		P value	OPD		ED		P value	OPD		ED		P value	OPD		ED		P value
	N	%	N	%		N	%	N	%		N	%	N	%		N	%	N	%		N	%	N	%	
	88,226	97.24	2,503	2.76		76,862	76.41	23,729	23.59		72,196	74.87	24,231	25.13		30,919	86.50	4,826	13.5		61,417	93.69	4,134	6.31	
Stage					<0.001					<0.001					<0.001					<0.001					<0.001
1	35,154	98.91	389	1.09		19,256	89.61	2233	10.39		15,542	90.28	1,673	9.72		2,820	91.41	265	8.59		14,713	97.44	386	2.56	
2	34,880	98.36	583	1.64		17,948	74.90	6016	25.1		3,471	82.08	758	17.92		13,470	91.62	1232	8.38		11,810	96.19	468	3.81	
3	12,879	96.99	400	3.01		22,903	75.27	7526	24.73		13,412	78.08	3,765	21.92		5,525	93.08	411	6.92		9,450	94.72	527	5.28	
4	5,313	82.45	1,131	17.55		16,755	67.81	7,954	32.19		39,771	68.80	18,035	31.2		9,104	75.73	2,918	24.27		25,444	90.24	2,753	9.76	
Stage					<0.001					<0.001					<0.001					<0.001					<0.001
Early	70,034	98.63	972	1.37		37,204	81.85	8249	18.15		19,013	88.66	2,431	11.34		16,290	91.58	1497	8.42		26,523	96.88	854	3.12	
Advanced	18,192	92.24	1,531	7.76		39,658	71.92	15,480	28.08		53,183	70.93	21,800	29.07		14,629	81.46	3,329	18.54		34,894	91.41	3,280	8.59	
Year of diagnosis					0.001					<0.001					<0.001					<0.001					0.167
2008	6,289	97.46	164	2.54		5,643	77.05	1,681	22.95		5,729	75.94	1,815	24.06		1,883	87.42	271	12.58		4,326	94.45	254	5.55	
2009	6,884	98.05	137	1.95		6,315	77.29	1,856	22.71		6,380	75.93	2,022	24.07		2,351	86.47	368	13.53		5,733	93.83	377	6.17	
2010	7,831	97.46	204	2.54		7,418	76.96	2,221	23.04		6,336	73.51	2,283	26.49		2,691	86.08	435	13.92		6,178	93.83	406	6.17	
2011	8,167	97.37	221	2.63		7,570	75.90	2,404	24.1		6,619	73.23	2,420	26.77		2,992	86.08	484	13.92		6,218	93.48	434	6.52	
2012	8,560	97.06	259	2.94		8,010	76.81	2,419	23.19		7,016	73.08	2,585	26.92		2,989	86.21	478	13.79		6,392	94.12	399	5.88	
2013	9,101	97.33	250	2.67		8,039	76.80	2,428	23.2		7,271	75.28	2,388	24.72		3,178	86.50	496	13.5		6,258	93.56	431	6.44	
2014	9,575	97.28	268	2.72		8,528	77.34	2,499	22.66		7,653	74.61	2,604	25.39		3,316	86.62	512	13.38		6,585	93.64	447	6.36	
2015	10,189	97.18	296	2.82		8,334	76.12	2,614	23.88		8,119	75.22	2,675	24.78		3,537	86.14	569	13.86		6,488	93.53	449	6.47	
2016	10,275	96.65	356	3.35		8,277	75.23	2,725	24.77		8,411	75.80	2,686	24.2		3,788	86.60	586	13.4		6,638	93.72	445	6.28	
2017	11,355	97.03	348	2.97		8,728	75.18	2,882	24.82		8,662	75.88	2,753	24.12		4,194	86.99	627	13.01		6,601	93.06	492	6.94	

ED, Emergency department.

To further explore each variable, logistic regression was performed to analyze differences in the risk of diagnosis through the emergency department for different types of cancer and its related factors (**Table 2**). Breast cancer was used as the reference. Compared with the risk of diagnosis through the emergency department for patients with breast cancer, the risk of diagnosis through that department was 8.4 times (95% confidence interval [95% CI, 7.99-8.78) higher for patients with lung cancer, 7.9 times (95% CI, 7.58-8.31) higher for those with colorectal cancer, 3.6 times (95% CI, 3.37-3.78) higher for those with prostate cancer, and 1.8 times (95% CI, 1.74-1.95) higher for those with oral cancer. The differences all reached statistical significance (P < 0.05). The probability of being diagnosed through the emergency department was significantly higher (P < 0.05) for male (odds ratio [OR], 1.18) patients older than 75 years old (OR, 1.22), those with a low level of education (OR, 1.30~1.75), those who were single (including unmarried, divorced, or widowed; OR, 1.25~1.51), those who earned a lower salary (OR, 1.12~1.52), and those with diabetes mellitus (OR, 1.37), chronic kidney disease (OR, 1.92), stroke (OR, 1.67), hypertension (OR, 1.35), or chronic mental illness (OR, 1.82).

**Table 2:** Bivariate analysis and multivariable logistic regression analysis of diagnoses made through the emergency and non-emergency departments and related factors for five cancers.

	Total		Non-emergency		Emergency		p value	aOR	95%CI		p value
	N	%	N	%	N	%					
Total	389,043	100	329,620	84.73	59,423	15.27					
Type of cancer							<0.001				
Female breast	90,729	23.32	88,226	97.24	2,503	2.76		1	–	–	–
Oral	65,551	16.85	61,417	93.69	4,134	6.31		1.29	1.22	1.37	<0.001
Prostate	35,745	9.19	30,919	86.5	4,826	13.5		2.54	2.39	2.69	<0.001
Lung	96,427	24.79	72,196	74.87	24,231	25.13		5.19	4.94	5.45	<0.001
Colorectal	100,591	25.86	76,862	76.41	23,729	23.59		6.49	6.19	6.8	<0.001
Stage							<0.001				
1	92,431	23.76	87,485	94.65	4,946	5.35		1	–	–	–
2	90,636	23.3	81,579	90.01	9,057	9.99		1.95	1.88	2.03	<0.001
3	76,798	19.74	64,169	83.56	12,629	16.44		2.37	2.28	2.45	<0.001
4	129,178	33.2	96,387	74.62	32,791	25.38		4.09	3.95	4.23	<0.001
Sex							<0.001				
Female	182,044	46.79	159,655	87.7	22,389	12.3		1	–	–	–
Male	206,999	53.21	169,965	82.11	37,034	17.89		1.15	1.12	1.17	<0.001
Age group							<0.001				
20-34	6,871	1.77	6,210	90.38	661	9.62		1	–	–	–
35-44	33,400	8.59	30,813	92.25	2,587	7.75		0.84	0.76	0.93	0.001
45-54	77,530	19.93	70,762	91.27	6,768	8.73		0.76	0.69	0.83	<0.001
55-64	97,529	25.07	86,445	88.64	11,084	11.36		0.69	0.63	0.76	<0.001
65-74	82,829	21.29	70,072	84.6	12,757	15.4		0.71	0.64	0.78	<0.001
≧75	90,884	23.36	65,318	71.87	25,566	28.13		1.14	1.03	1.25	0.009
Educational level							<0.001				
Elementary school and below	142,010	36.5	112,879	79.49	29,131	20.51		1.63	1.58	1.69	<0.001
Junior high	65,959	16.95	56,853	86.19	9,106	13.81		1.47	1.42	1.53	<0.001
Senior high	93,882	24.13	83,467	88.91	10,415	11.09		1.24	1.2	1.28	<0.001
University and above	76,646	19.7	69,875	91.17	6,771	8.83		1	–	–	–
Missing value	10,546	2.71		0		0					
Marital status							<0.001				
Unmarried	30,328	7.8	26,210	86.42	4,118	13.58		1.42	1.36	1.48	<0.001
Married	262,682	67.52	228,022	86.81	34,660	13.19		1	–	–	–
Divorced	34,372	8.84	29,684	86.36	4,688	13.64		1.24	1.19	1.28	<0.001
Widowed	51,094	13.13	39,132	76.59	11,962	23.41		1.2	1.17	1.24	<0.001
Missing value	10,567	2.72		0		0					
Monthly Salary (TWD)							<0.001				
≦17,280	97,737	25.12	79,455	81.29	18,282	18.71		1.38	1.3	1.47	<0.001
17,281-22,800	146,367	37.62	122,274	83.54	24,093	16.46		1.2	1.13	1.27	<0.001
22,801-28,800	26,434	6.79	23,225	87.86	3,209	12.14		1.16	1.08	1.25	<0.001
28,801-36,300	33,173	8.53	29,207	88.04	3,966	11.96		1.13	1.06	1.21	<0.001
36,301-45,800	39,711	10.21	35,047	88.26	4,664	11.74		1.1	1.03	1.18	0.004
45,801-57,800	15,141	3.89	13,320	87.97	1,821	12.03		1.09	1	1.17	0.041
57,801-72,800	14,413	3.7	12,723	88.27	1,690	11.73		1.07	0.99	1.16	0.093
≧72,801	16,067	4.13	14,369	89.43	1,698	10.57		1	–	–	–
Degree of urbanization							<0.001				
1	107,828	27.72	92,209	85.51	15,619	14.49		1	–	–	–
2	121,231	31.16	104,065	85.84	17,166	14.16		0.92	0.89	0.94	<0.001
3	64,030	16.46	54,283	84.78	9,747	15.22		0.91	0.88	0.94	<0.001
4	54,412	13.99	45,148	82.97	9,264	17.03		0.95	0.92	0.99	0.006
5	9,450	2.43	7,649	80.94	1,801	19.06		0.92	0.87	0.98	0.011
6	16,409	4.22	13,374	81.5	3,035	18.5		0.96	0.91	1.01	0.074
7	15,683	4.03	12,892	82.2	2,791	17.8		0.94	0.89	0.99	0.023
Severity of comorbidities							<0.001				
0 point	337,733	86.81	283,761	84.02	53,972	15.98		1	–	–	–
1 point	37,449	9.63	34,024	90.85	3,425	9.15		0.55	0.52	0.57	<0.001
2 points	9,272	2.38	8,004	86.32	1,268	13.68		0.65	0.61	0.69	<0.001
≧3 points	4,589	1.18	3,831	83.48	758	16.52		0.69	0.63	0.76	<0.001
Diabetes mellitus							<0.001				
No	360,640	92.7	309,470	85.81	51,170	14.19		1	–	–	–
Yes	28,403	7.3	20,150	70.94	8,253	29.06		1.38	1.34	1.43	<0.001
Chronic kidney disease							<0.001				
No	386,090	99.24	327,742	84.89	58,348	15.11		1	–	–	–
Yes	2,953	0.76	1,878	63.6	1,075	36.4		2.03	1.86	2.21	<0.001
Stroke							<0.001				
No	378,998	97.42	323,118	85.26	55,880	14.74		1	–	–	–
Yes	10,045	2.58	6,502	64.73	3,543	35.27		1.66	1.58	1.75	<0.001
Hypertension							<0.001				
No	339,981	87.39	293,797	86.42	46,184	13.58		1	–	–	–
Yes	49,062	12.61	35,823	73.02	13,239	26.98		1.41	1.37	1.44	<0.001
Chronic mental illness							<0.001				
No	382,793	98.39	325,365	85	57,428	15		1	–	–	–
Yes	6,250	1.61	4,255	68.08	1,995	31.92		1.82	1.71	1.93	<0.001
Level of main medical institution attended							<0.001				
Medical center	63,795	16.4	53,086	83.21	10,709	16.79		1	–	–	–
Regional hospital	73,324	18.85	60,726	82.82	12,598	17.18		0.93	0.9	0.96	0.002
District hospital	40,056	10.3	33,424	83.44	6,632	16.56		0.85	0.81	0.88	<0.001
Clinic	206,319	53.03	177,707	86.13	28,612	13.87		0.83	0.8	0.85	<0.001
Missing value	5,549	1.43		0		0					
Type of ownership							<0.001				
Nonpublic	325,639	83.7	276,570	84.93	49,069	15.07		1	–	–	–
Public	62,164	15.98	51,810	83.34	10,354	16.66		0.9	0.88	0.93	<0.001
Missing value	1,240	0.32		0		0					
Year of diagnosis							<0.001				
2008	28,055	7.21	23,870	85.08	4,185	14.92		1	–	–	–
2009	32,423	8.33	27,663	85.32	4,760	14.68		1.02	0.97	1.07	0.531
2010	36,003	9.25	30,454	84.59	5,549	15.41		1.09	1.04	1.15	<0.001
2011	37,529	9.65	31,566	84.11	5,963	15.89		1.03	0.98	1.08	0.307
2012	39,107	10.05	32,967	84.3	6,140	15.7		1.04	0.99	1.09	0.12
2013	39,840	10.24	33,847	84.96	5,993	15.04		1.1	1.05	1.16	<0.001
2014	41,987	10.79	35,657	84.92	6,330	15.08		1.12	1.07	1.18	<0.001
2015	43,270	11.12	36,667	84.74	6,603	15.26		1.16	1.11	1.21	<0.001
2016	44,187	11.36	37,389	84.62	6,798	15.38		1.19	1.13	1.24	<0.001
2017	46,642	11.99	39,540	84.77	7,102	15.23		1.17	1.12	1.23	<0.001

CI, confidence interval; OR, odds ratio; aOR, adjusted odds ratio; TWD, Taiwan Dollar.

Overall, approximately 47.1% of participants had early-stage cancer and 52.9% had advanced-stage cancer (**Table 3**). Among those diagnosed through emergency departments, approximately 76.4% had advanced cancer. The proportion of diagnosis of early-stage cancer was the highest for female breast cancer at 78.3%, while the proportion of diagnosis of advanced cancer was the highest for lung cancer at 77.8%. Patients with cancer who were male, older, and had lower education levels were more likely to be diagnosed with advanced cancer. In the health status analysis, the proportion of diagnosis with advanced cancer was the highest for newly diagnosed patients with a comorbidity severity (CCI) of 3 points or more (57.7%), followed by patients with 2 points (53.7%). The proportion of patients diagnosed with advanced cancer was also high for patients with diabetes mellitus, stroke, hypertension, or chronic mental illness, and the difference was statistically significant (P < 0.05).

**Table 3:** Bivariate analysis and multivariable logistic regression analysis of the correlation between cancer stage at diagnosis and route to diagnosis for patients with one of the five cancers.

	Total		Early stage		Advanced stage		p value	aOR	95%CI		p value
	N	%	N	%	N	%					
Total	389,043	100	183,067	47.06	205,976	52.94					
Type of cancer							<0.001				
Female breast	90,729	23.32	71,006	78.26	19,723	21.74		1			
Prostate	35,745	9.19	17,787	49.76	17,958	50.24		2.84	2.75	2.94	<0.001
Oral	65,551	16.85	27,377	41.76	38,174	58.24		3.18	3.11	3.26	<0.001
Colorectal	100,591	25.86	45,453	45.19	55,138	54.81		3.83	3.73	3.94	<0.001
Lung	96,427	24.79	21,444	22.24	74,983	77.76		9.08	8.85	9.32	<0.001
Route to diagnosis							<0.001				
Non-emergency	329,620	84.73	169,064	51.29	160,556	48.71		1			
Emergency	59,423	15.27	14,003	23.56	45,420	76.44		2.24	2.19	2.29	<0.001
Sex							<0.001				
Female	182,044	46.79	105,329	57.86	76,715	42.14		1			
Male	206,999	53.21	77,738	37.55	129,261	62.45		1.26	1.24	1.29	<0.001
Age group							<0.001				
20-34	6,871	1.77	4,078	59.35	2,793	40.65		1			
35-44	33,400	8.59	19,357	57.96	14,043	42.04		1.15	1.09	1.22	<0.001
45-54	77,530	19.93	42,115	54.32	35,415	45.68		1.21	1.14	1.28	<0.001
55-64	97,529	25.07	48,167	49.39	49,362	50.61		1.16	1.09	1.23	<0.001
65-74	82,829	21.29	36,858	44.5	45,971	55.5		1.11	1.05	1.18	0.001
≧75	90,884	23.36	32,492	35.75	58,392	64.25		1.27	1.19	1.35	<0.001
Educational level							<0.001				
Elementary school and below	142,010	36.5	58,225	41	83,785	59		1			
Junior high	65,959	16.95	30,292	45.93	35,667	54.07		1.37	1.34	1.41	<0.001
Senior high	93,882	24.13	48,122	51.26	45,760	48.74		1.29	1.26	1.33	<0.001
University and above	76,646	19.7	45,210	58.99	31,436	41.01		1.2	1.17	1.23	<0.001
Missing value	10,546	2.71	1,218	11.55	9,328	88.45					
Marital status							<0.001				
Unmarried	30,328	7.8	15,077	49.71	15,251	50.29		1.33	1.29	1.37	<0.001
Married	262,682	67.52	129,831	49.43	132,851	50.57		1			
Divorced	34,372	8.84	15,677	45.61	18,695	54.39		1.22	1.19	1.25	<0.001
Widowed	51,094	13.13	21,242	41.57	29,852	58.43		1.19	1.16	1.22	<0.001
Missing value	10,567	2.72	1,240	11.73	9,327	88.27					
Monthly salary (TWD)							<0.001				
≦17,280	97,737	25.12	41,502	42.46	56,235	57.54		1.53	1.47	1.59	<0.001
17,281-22,800	146,367	37.62	64,955	44.38	81,412	55.62		1.36	1.31	1.41	<0.001
22,801-28,800	26,434	6.79	13,318	50.38	13,116	49.62		1.37	1.31	1.43	<0.001
28,801-36,300	33,173	8.53	16,989	51.21	16,184	48.79		1.32	1.26	1.38	<0.001
36,301-45,800	39,711	10.21	20,928	52.7	18,783	47.3		1.22	1.17	1.27	<0.001
45,801-57,800	15,141	3.89	8,018	52.96	7,123	47.04		1.27	1.21	1.33	<0.001
57,801-72,800	14,413	3.7	7,929	55.01	6,484	44.99		1.18	1.13	1.25	<0.001
≧72,801	16,067	4.13	9,428	58.68	6,639	41.32		1			
Degree of urbanization							<0.001				
1	107,828	27.72	54,954	50.96	52,874	49.04		1			
2	121,231	31.16	58,861	48.55	62,370	51.45		1.04	1.02	1.06	<0.001
3	64,030	16.46	29,079	45.41	34,951	54.59		1.08	1.06	1.11	<0.001
4	54,412	13.99	23,658	43.48	30,754	56.52		1.07	1.05	1.1	<0.001
5	9,450	2.43	3,668	38.81	5,782	61.19		1.09	1.04	1.15	0.001
6	16,409	4.22	6,428	39.17	9,981	60.83		1.11	1.07	1.16	<0.001
7	15,683	4.03	6,419	40.93	9,264	59.07		1.08	1.03	1.12	<0.001
Severity of comorbidities							<0.001				
0 point	337,733	86.81	158,394	46.9	179,339	53.1		1			
1 point	37,449	9.63	18,441	49.24	19,008	50.76		0.84	0.82	0.86	<0.001
2 points	9,272	2.38	4,289	46.26	4,983	53.74		0.8	0.77	0.84	<0.001
≧3 points	4,589	1.18	1,943	42.34	2,646	57.66		0.88	0.82	0.94	<0.001
Diabetes mellitus							<0.001				
No	360,640	92.7	171,750	47.62	188,890	52.38		1			
Yes	28,403	7.3	11,317	39.84	17,086	60.16		0.99	0.96	1.02	0.373
Chronic kidney disease							0.385				
No	386,090	99.24	181,840	47.1	204,250	52.9		1			
Yes	2,953	0.76	1,227	41.55	1,726	58.45		0.78	0.72	0.85	<0.001
Stroke							<0.001				
No	378,998	97.42	179,547	47.37	199,451	52.63		1			
Yes	10,045	2.58	3,520	35.04	6,525	64.96		1.09	1.04	1.14	0.001
Hypertension							<0.001				
No	339,981	87.39	162,676	47.85	177,305	52.15		1			
Yes	49,062	12.61	20,391	41.56	28,671	58.44		0.84	0.82	0.86	<0.001
Chronic mental illness							<0.001				
No	382,793	98.39	180,686	47.2	202,107	52.8		1			
Yes	6,250	1.61	2,381	38.1	3,869	61.9		1.07	1.01	1.13	0.029
Level of main medical institution attended							<0.001				
Medical center	63,795	16.4	31,493	49.37	32,302	50.63		1			
Regional hospital	73,324	18.85	34,161	46.59	39,163	53.41		1.1	1.08	1.13	<0.001
District hospital	40,056	10.3	18,018	44.98	22,038	55.02		1.16	1.12	1.19	<0.001
Clinic	206,319	53.03	97,282	47.15	109,037	52.85		1.23	1.2	1.25	<0.001
Missing value	5,549	1.43	2,113	38.08	3,436	61.92					
Type of ownership							0.045				
Nonpublic	325,639	83.7	153,226	47.05	172,413	52.95		1			
Public	62,164	15.98	29,523	47.49	32,641	52.51		0.95	0.93	0.97	<0.001
Missing value	1,240	0.32	318	25.65	922	74.35					
Year of diagnosis							<0.001				
2008	28,055	7.21	12,095	43.11	15,960	56.89		1			
2009	32,423	8.33	14,441	44.54	17,982	55.46		0.93	0.89	0.96	<0.001
2010	36,003	9.25	16,388	45.52	19,615	54.48		0.9	0.87	0.93	<0.001
2011	37,529	9.65	16,967	45.21	20,562	54.79		0.83	0.8	0.86	<0.001
2012	39,107	10.05	18,169	46.46	20,938	53.54		0.79	0.76	0.82	<0.001
2013	39,840	10.24	18,834	47.27	21,006	52.73		0.86	0.83	0.89	<0.001
2014	41,987	10.79	20,091	47.85	21,896	52.15		0.84	0.81	0.87	<0.001
2015	43,270	11.12	21,114	48.8	22,156	51.2		0.81	0.79	0.84	<0.001
2016	44,187	11.36	21,464	48.58	22,723	51.42		0.82	0.79	0.85	<0.001
2017	46,642	11.99	23,504	50.39	23,138	49.61		0.75	0.72	0.77	<0.001

CI, confidence interval; OR, odds ratio; aOR, adjusted odds ratio; TWD, Taiwan Dollar.

The impact of the route to diagnosis on the diagnosis of advanced cancer was explored (**Table 3**) through logistic regression analysis. After controlling for relevant variables, the results showed that patients with cancer diagnosed through an emergency department were more likely to be diagnosed with advanced cancer than those diagnosed through a nonemergency setting (OR, 2.24; 95%CI, 2.19–2.29). After controlling for the effect of the route to diagnosis, the probability of patients with any of the other four cancers being diagnosed with advanced cancer was 3.2 to 9.1 times higher than that of patients with breast cancer, with the highest probability observed for patients with lung cancer (OR, 9.1). All differences were statistically significant (P < 0.05).

In total, 389,043 patients were newly diagnosed with cancer from 2008 to 2017. By the end of 2018, 164,949 patients had died (**Table 4**). Among them, the highest number of deaths was observed for patients with lung cancer (71,171 [73.8%]), while the lowest mortality rate was observed for patients with breast cancer (14.2%). Of the patients with cancer diagnosed through the emergency department, 42,165 (71.0%) patients died. Until 2018, the mortality rate was highest for patients diagnosed with stage 4 cancer (approximately 77.7%).

**Table 4:** Long rank tests and Cox proportional hazards models of factors associated with survival among patients with one of the five cancers.

	Total		Survival		Death		P value	aHR	95%CI		p value
	N	%	N	%	N	%					
	389,043	100	224,094	57.6	164,949	42.4					
Type of cancer							<0.001				
Female breast	90,729	23.32	77,831	85.78	12,898	14.22		1			
Prostate	35,745	9.19	25,079	70.16	10,666	29.84		0.48	0.46	0.49	<0.001
Oral	65,551	16.85	38,737	59.09	26,814	40.91		1.11	1.08	1.14	<0.001
Colorectal	100,591	25.86	57,191	56.85	43,400	43.15		1.37	1.34	1.4	<0.001
Lung	96,427	24.79	25,256	26.19	71,171	73.81		2.64	2.58	2.7	<0.001
Route to diagnosis							<0.001				
Non-emergency	329,620	84.73	206,836	62.75	122,784	37.25		1			
Emergency	59,423	15.27	17,258	29.04	42,165	70.96		1.46	1.44	1.48	<0.001
Stage							<0.001				
stage 1	92,431	23.76	80,799	87.42	11,632	12.58		1			
stage 2	90,636	23.3	71,604	79	19,032	21		1.87	1.82	1.91	<0.001
stage 3	76,798	19.74	42,889	55.85	33,909	44.15		3.58	3.51	3.66	<0.001
stage 4	129,178	33.2	28,802	22.3	100,376	77.7		9.33	9.14	9.52	<0.001
Sex							<0.001				
Female	182,044	46.79	123,620	67.91	58,424	32.09		1			
Male	206,999	53.21	100,474	48.54	106,525	51.46		1.56	1.54	1.58	<0.001
Age group							<0.001				
20-34	6,871	1.77	5,246	76.35	1,625	23.65		1			
35-44	33,400	8.59	24,748	74.1	8,652	25.9		1.16	1.09	1.22	<0.001
45-54	77,530	19.93	54,373	70.13	23,157	29.87		1.23	1.16	1.29	<0.001
55-64	97,529	25.07	63,689	65.3	33,840	34.7		1.31	1.24	1.38	<0.001
65-74	82,829	21.29	46,158	55.73	36,671	44.27		1.64	1.55	1.73	<0.001
≧75	90,884	23.36	29,880	32.88	61,004	67.12		2.76	2.61	2.91	<0.001
Educational level							<0.001				
Elementary school and below	142,010	36.5	65,211	45.92	76,799	54.08		1			
Junior high	65,959	16.95	39,007	59.14	26,952	40.86		1.37	1.35	1.39	<0.001
Senior high	93,882	24.13	62,405	66.47	31,477	33.53		1.28	1.25	1.3	<0.001
University and above	76,646	19.7	56,972	74.33	19,674	25.67		1.16	1.14	1.18	<0.001
Missing value	10,546	2.71		0		0					
Marital status							<0.001				
Unmarried	30,328	7.8	19,184	63.26	11,144	36.74		1.31	1.28	1.34	<0.001
Married	262,682	67.52	161,357	61.43	101,325	38.57		1			
Divorced	34,372	8.84	20,689	60.19	13,683	39.81		1.21	1.19	1.24	<0.001
Widowed	51,094	13.13	22,342	43.73	28,752	56.27		1.22	1.21	1.24	<0.001
Missing value	10,567	2.72		0		0					
Monthly salary (TWD)							<0.001				
	≦17,280	97,737	25.12	48,839	49.97	48,898	50.03	1.25	1.21	1.29	<0.001
17,281-22,800	146,367	37.62	79,161	54.08	67,206	45.92		1.14	1.1	1.18	<0.001
22,801-28,800	26,434	6.79	16,746	63.35	9,688	36.65		1.13	1.09	1.18	<0.001
28,801-36,300	33,173	8.53	21,600	65.11	11,573	34.89		1.1	1.07	1.14	<0.001
36,301-45,800	39,711	10.21	26,450	66.61	13,261	33.39		1.07	1.03	1.11	0
45,801-57,800	15,141	3.89	10,065	66.48	5,076	33.52		1.05	1	1.09	0.035
57,801-72,800	14,413	3.7	9,633	66.84	4,780	33.16		1.05	1.01	1.1	0.016
≧72,801	16,067	4.13	11,600	72.2	4,467	27.8		1			
	389,043	100	224,094	57.6	164,949	42.4					
Degree of urbanization							<0.001				
1	107,828	27.72	66,853	62	40,975	38		1			
2	121,231	31.16	72,443	59.76	48,788	40.24		1.02	1.01	1.03	0.005
3	64,030	16.46	36,254	56.62	27,776	43.38		1.04	1.02	1.06	<0.001
4	54,412	13.99	28,693	52.73	25,719	47.27		1.04	1.02	1.05	<0.001
5	9,450	2.43	4,351	46.04	5,099	53.96		1.03	1	1.06	0.07
6	16,409	4.22	7,667	46.72	8,742	53.28		1.09	1.06	1.11	<0.001
7	15,683	4.03	7,833	49.95	7,850	50.05		1.06	1.03	1.09	<0.001
Severity of comorbidities							<0.001				
0 point	337,733	86.81	194,936	57.72	142,797	42.28		1			
1 point	37,449	9.63	22,666	60.52	14,783	39.48		0.92	0.91	0.94	<0.001
2 points	9,272	2.38	4,640	50.04	4,632	49.96		1.05	1.02	1.08	0.002
≧3 points	4,589	1.18	1,852	40.36	2,737	59.64		1.19	1.15	1.24	<0.001
Diabetes mellitus							<0.001				
No	360,640	92.7	212,734	58.99	147,906	41.01		1			
Yes	28,403	7.3	11,360	40	17,043	60		1.21	1.19	1.23	<0.001
Chronic kidney disease							<0.001				
No	386,090	99.24	223,237	57.82	162,853	42.18		1			
Yes	2,953	0.76	857	29.02	2,096	70.98		2.15	2.05	2.25	<0.001
Stroke							<0.001				
No	378,998	97.42	221,175	58.36	157,823	41.64		1			
Yes	10,045	2.58	2,919	29.06	7,126	70.94		1.34	1.3	1.37	<0.001
Hypertension							<0.001				
No	339,981	87.39	202,270	59.49	137,711	40.51		1			
Yes	49,062	12.61	21,824	44.48	27,238	55.52		0.97	0.95	0.98	<0.001
Chronic mental illness							<0.001				
No	382,793	98.39	221,953	57.98	160,840	42.02		1			
Yes	6,250	1.61	2,141	34.26	4,109	65.74		1.46	1.41	1.5	<0.001
Level of main medical institution attended							<0.001				
Medical center	63,795	16.4	36,366	57	27,429	43		1			
Regional hospital	73,324	18.85	40,068	54.65	33,256	45.35		1.04	1.02	1.06	<0.001
District hospital	40,056	10.3	21,340	53.28	18,716	46.72		1	0.98	1.02	0.956
Clinic	206,319	53.03	123,674	59.94	82,645	40.06		0.95	0.93	0.96	<0.001
Missing value	5,549	1.43		0		0					
Type of ownership							<0.001				
Nonpublic	325,639	83.7	189,601	58.22	136,038	41.78		1			
Public	62,164	15.98	33,994	54.68	28,170	45.32		0.95	0.94	0.97	<0.001
Missing value	1,240	0.32		0		0					
Year of diagnosis							<0.001				
2008	28,055	7.21	11,280	40.21	16,775	59.79		1			
2009	32,423	8.33	14,182	43.74	18,241	56.26		0.96	0.94	0.98	<0.001
2010	36,003	9.25	16,967	47.13	19,036	52.87		0.92	0.9	0.94	<0.001
2011	37,529	9.65	18,374	48.96	19,155	51.04		0.75	0.73	0.77	<0.001
2012	39,107	10.05	20,620	52.73	18,487	47.27		0.71	0.7	0.73	<0.001
2013	39,840	10.24	22,240	55.82	17,600	44.18		0.87	0.85	0.89	<0.001
2014	41,987	10.79	25,003	59.55	16,984	40.45		0.86	0.84	0.88	<0.001
2015	43,270	11.12	27,704	64.03	15,566	35.97		0.85	0.83	0.87	<0.001
2016	44,187	11.36	30,877	69.88	13,310	30.12		0.81	0.79	0.83	<0.001
2017	46,642	11.99	36,847	79	9,795	21		0.67	0.65	0.69	<0.001

CI, confidence interval; HR, hazard ratio; aHR, adjusted hazard ratio; TWD, Taiwan Dollar.

**Figure 4:**
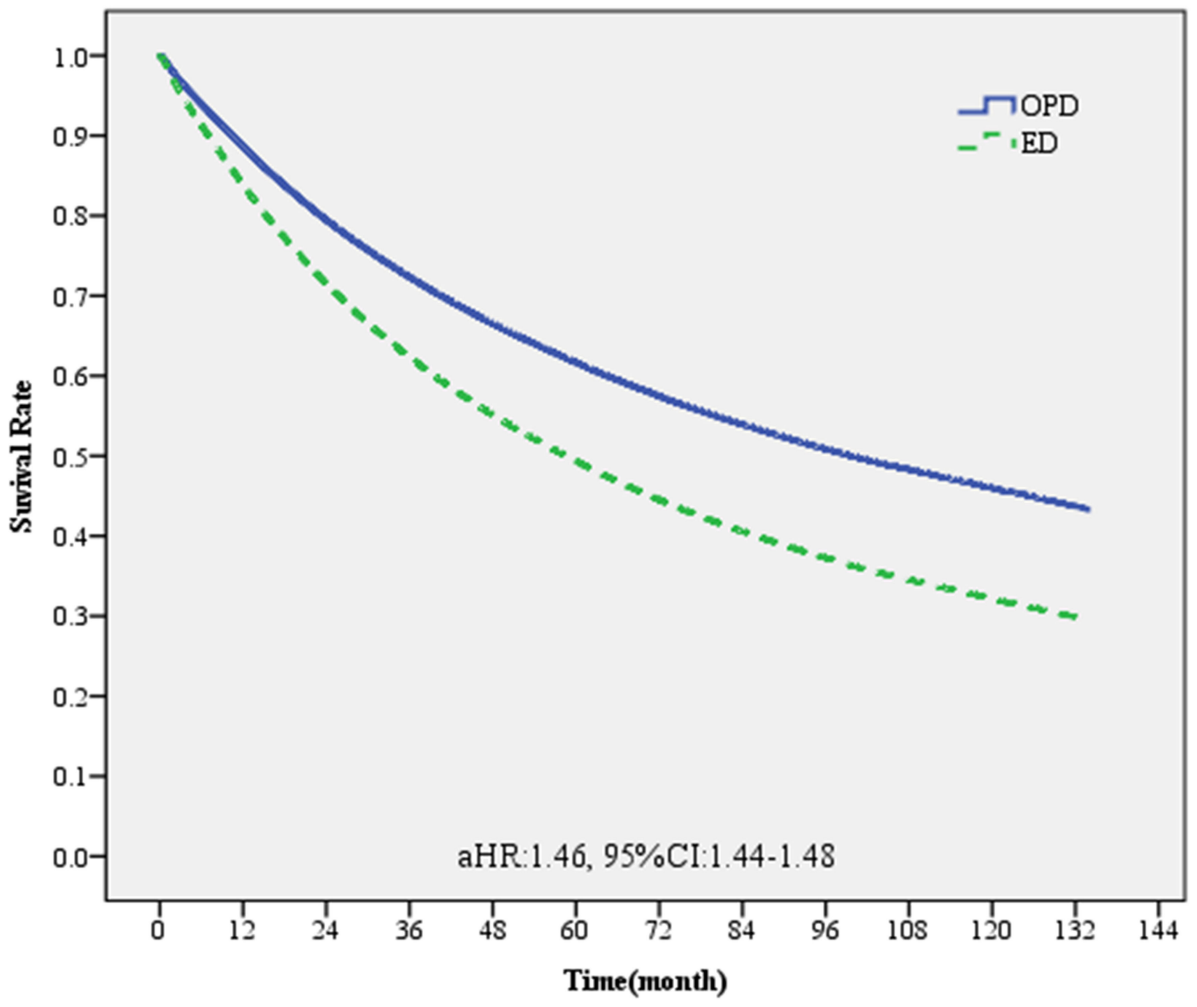
Survival curve of patients with one of the five major cancers diagnosed through emergency and non-emergency departments.

The Cox proportional hazard model was adopted in the current study, and patients with cancer were tracked from the time of diagnosis until death or the end of 2018 (**Table 4**). Patients diagnosed with cancer through emergency departments had a significantly higher risk of death (aHR, 1.46; 95% CI, 1.44-1.48) (**Figure 4**). Compared with breast cancer, prostate cancer had a significantly lower risk of mortality (aHR, 0.48; 95% CI, 0.46-0.49), while the risk of mortality for oral (aHR, 1.11; 95% CI, 1.08-1.14), colorectal (aHR, 1.37; 95% CI, 1.34-1.40), and lung (aHR, 2.64; 95% CI, 2.58-2.70) cancers were all significantly higher (P < 0.05). Cancer stage, baseline personal characteristics, environment, economic factors, health status, type of main medical institution, and the year of diagnosis all were significantly associated with the survival of patients with cancer.

We further conducted stratified analyses for each cancer type. The results showed that patients with prostate cancer diagnosed at the emergency department had the highest death risk compared to those diagnosed at the non-emergency department (aHR, 1.76; 95%CI, 1.68–1.84), followed by patients with breast cancer (aHR, 1.62; 95%CI, 1.52-1.73) (**Table 5**).

**Table 5:** Relative mortality risks associated with the route to diagnosis and cancer staging for each cancer.

	Breast cancer				Prostate cancer				Oral cancer				Colon cancer				Lung cancer			
	aHR	95%CI		p value	aHR	95%CI		p value	aHR	95%CI		p value	aHR	95%CI		p value	aHR	95%CI		p value
Route to diagnosis																				
Non-emergency	1.00				1.00				1.00				1.00				1.00			
Emergency	1.62	1.52	1.73	<0.001	1.76	1.68	1.84	<0.001	1.59	1.52	1.66	<0.001	1.43	1.40	1.46	<0.001	1.41	1.38	1.43	<0.001
Cancer stage																				
1	1.00				1.00				1.00				1.00				1.00			
2	2.56	2.40	2.73	<0.001	1.30	1.16	1.45	<0.001	1.48	1.41	1.56	<0.001	1.54	1.48	1.61	<0.001	2.66	2.52	2.81	<0.001
3	7.29	6.84	7.77	<0.001	1.31	1.16	1.48	<0.001	2.24	2.13	2.36	<0.001	2.54	2.45	2.64	<0.001	5.54	5.33	5.76	<0.001
4	28.66	26.86	30.58	<0.001	5.11	4.59	5.68	<0.001	4.75	4.56	4.95	<0.001	11.82	11.39	12.28	<0.001	9.59	9.25	9.94	<0.001

The above models were performed with Cox proportional hazards models and have been controlled for variables including gender, age, educational level, marital status, monthly salary, degree of urbanization, severity of comorbidities, diabetes mellitus, chronic kidney disease, hypertension, chronic mental illness, level of main medical institution attended, type of ownership, and year of diagnosis.

**Figure 5:**
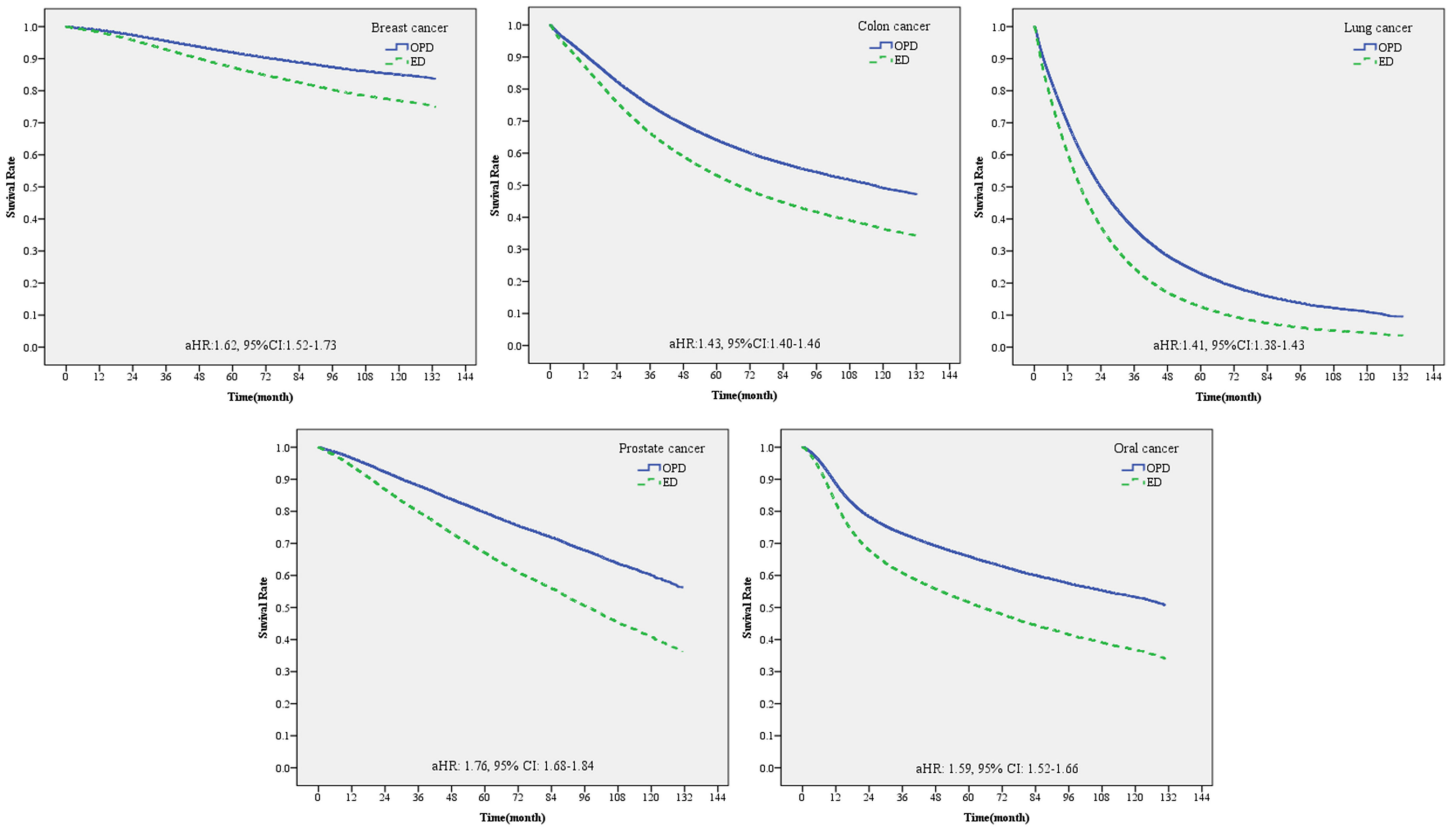
Survival curve of patients with different cancers diagnosed through emergency and non-emergency departments.

Finally, whether the 5-year cancer survival rate was different between patients diagnosed through the emergency and non-emergency departments was explored (**Table 6**; **Figure 5**). The overall 5-year survival rate of patients with any of the five cancers included in this study was approximately 56.7%. Among them, the 5-year survival rate of patients diagnosed with cancer through the emergency department was only 26.9%. The survival rate of patients with stage 1 cancer was the highest (88.0%), while the survival rate of those with stage 4 cancer was the lowest (18.9%). In terms of cancer type, the 5-year survival rate of patients with breast cancer was the highest at 86.0%, while that of patients with breast cancer diagnosed through the emergency department was 49.3%. The 5-year survival rate of patients with lung cancer was the lowest (22.2%), while that of patients with lung cancer diagnosed through the emergency department was 8.87%. For patients diagnosed with stage 4 lung cancer through the emergency department, the 5-year survival rate was even lower (3.51%).

**Table 6:** Five-year survival rate of patients with one of the five cancers by route to diagnosis.

	Total	Non-emergency department	Emergency department
Breast cancer	86.04%	87.06%	49.25%
stage 1	96.78%	96.88%	87.53%
stage 2	90.44%	90.61%	79.59%
stage 3	73.41%	74.05%	51.85%
stage 4	29.43%	31.77%	18.05%
Colon cancer	55.97%	61.44%	38.19%
stage 1	84.33%	87.08%	60.83%
stage 2	71.87%	75.95%	59.65%
stage 3	59.49%	64.01%	45.62%
stage 4	11.52%	13.21%	7.95%
Lung cancer	22.18%	26.68%	8.87%
stage 1	77.99%	80.84%	52.53%
stage 2	43.98%	46.56%	32.16%
stage 3	17.52%	19.48%	10.51%
stage 4	6.34%	7.60%	3.51%
Prostate cancer	70.54%	74.95%	42.13%
stage 1	87.92%	88.74%	79.33%
stage 2	83.77%	85.64%	63.22%
stage 3	85.62%	87.09%	66.19%
stage 4	42.16%	47.26%	25.99%
Oral cancer	59.65%	61.32%	34.72%
stage 1	83.18%	83.41%	74.37%
stage 2	75.24%	75.93%	57.69%
stage 3	65.39%	66.44%	46.40%
stage 4	38.11%	39.74%	22.80%

## Discussion

The routes to diagnose cancer can be divided into three categories: cancer screening, outpatient clinics, and the emergency department (16–18). In the present study, the proportion of initial diagnoses made through the emergency department was the lowest for patients with breast cancer. This may be because noting abnormalities in the appearance of the breasts is easy, resulting in early stages being found at initial diagnosis and fewer diagnoses being made through the emergency department. The proportion of diagnoses made through the emergency department was highest for patients with lung cancer (25.1%), followed by that for patients with colorectal cancer (23.6%). This finding aligns with a SEER-Medicare study that showed EDs play a crucial role in diagnosing cancer in older US adults. ED visits accounted for 23% of cancer diagnoses among 614,748 patients, with the highest rates in colorectal and lung cancers (19). Despite variations in diagnostic standards, both lung cancer and rectal cancer impact patients’ lives in a significant manner. The former manifests as dyspnea or cough, while the latter leads to intestinal obstruction or bleeding, prompting patients to seek emergency treatment. Nonetheless, both cancers have room for tumor growth, making detection challenging. Hence, the onset of symptoms may necessitate emergency treatment, thereby increasing opportunities for cancer diagnosis under such circumstances.

However, a study by McPhail et al., which looked at eight types of cancer across six countries, contradicts our findings. The study includes data from 857,068 patients across 14 regions. The results showed that emergency diagnosis occurred in 24% to 42.5% of cases. The highest rate of emergency diagnosis was recorded in relation to pancreatic cancer, ranging from 34.1% to 60.4%. Conversely, the lowest rate was observed in rectal cancer cases, which ranged from 9.1% to 19.8% (20). There are various factors that could impact the percentage of emergency diagnoses, such as public perceptions and awareness regarding potential cancer symptoms, healthcare system organization, presence and participation in population-based cancer screening programs, and the type of definition used, whether broad or narrow. Furthermore, there may be other unmeasured variables that impact these findings (20).

In this study, the date of cancer diagnosis of the study participants was obtained through the cancer registry files. The use of cancer registration files to identify cancer is rarely used in other studies as a diagnosis of cancer. In addition, in the absence of any medical records for target cancer within 180 days before the diagnosis date, we determine whether there is any emergency department care record within two weeks (14 days) before the cancer diagnosis date to classify it as an emergency diagnosis. If no such records existed, they fall under the non-emergency diagnosis category. A patient’s cancer diagnosis occurred within 14 days after the emergence visit, and it was still considered an emergency-diagnosed cancer since the cancer-related diagnosis codes might be reported late due to the cancer-related examination process. Some studies defined an emergency diagnosis of cancer based on an emergency visit within 30 days before the diagnosis (19–21), for that definition is too imprecise and too much interference. Our definition is more precise, making it less prone to errors.

Since 1995, Taiwan has successively implemented services to screen for the four major cancers. Currently, free screening services are provided to the public for cervical, oral, breast, and colorectal cancers. According to the statistical analyses of the Health Promotion Administration of the Ministry of Health and Welfare of Taiwan, having a fecal occult blood test every 2 years may reduce the mortality rate of colorectal cancer by 23% and having a mammography examination every 2 years may reduce the mortality rate of breast cancer by 41%; regular examination of the oral mucosa may reduce the risk of death by 26% in men who chew betel nut or smoke (22). In the current study, stage 4 cancer was most common among patients diagnosed with cancer through the emergency department. Among all types of cancer, the highest proportion of stage 4 cancer was observed for patients with colorectal cancer (32.2%), followed by patients with lung cancer (31.2%). The lowest proportion of stage 4 cancer was observed among patients with breast cancer (17.6%). An analysis of cases by year showed that the number of diagnoses of various types of cancer through the emergency department has not decreased, even though the amount of screening data has increased each year. This discrepancy may result from that fact that most of the cases diagnosed through screening were in the early stages of disease. However, in the current study, approximately 76.4% of patients diagnosed with cancer through the emergency department had advanced cancer. Therefore, screening could not have reduced the proportion of diagnoses made through the emergency department.

Gender, age, education level, salary, degree of urbanization of the place of residence, medical insurance, family health history, and personal medical history are factors associated with the cancer stage at diagnosis (23–30). The cancer stage at diagnosis is generally higher for men than for women (23, 24). Similar findings were noted in the present study. Moreover, the cancer stage at diagnosis increases with increasing age (23, 25, 26). A study on prostate cancer showed that patients with advanced cancer at diagnosis were between 70 and 79 years old (27). Recently, a lot of studies reported that older patients with cancer were significantly more likely to be diagnosed with advanced cancer (19, 28). In Denmark, a study examined diagnosis routes for different age groups among 137,876 cancer patients. Most middle-aged patients were identified through cancer patient pathways, but younger and older patients were less likely to be diagnosed this way. Instead, more older patients were diagnosed via unplanned admission, death certificate, or outpatient admission (31). Our study indicated that a high proportion of patients over 75 years old were diagnosed with cancer through the emergency department or with advanced cancer. The relative lack of knowledge on cancer among older people, as well as their inability to obtain timely medical care, may cause a delay in their receiving care, thus resulting in the advanced cancer stage at diagnosis (29).

Cancer stage at diagnosis is more advanced for people with lower education levels than for those with higher education levels (7, 19, 20, 30), with lower income levels than for those with higher income levels (7, 22, 23, 27, 30), and for residents of rural areas than for residents of urban centers (7, 23, 30). Similarly, the findings of the current study revealed that patients were significantly more likely to be diagnosed with advanced cancer or die if they had low education levels, low salaries, or lived in suburban areas.

The literature suggests that unmarried individuals may be more likely to have their cancer diagnosed through emergency presentation, which can lead to poorer prognoses (19, 21). This could be due to a range of factors, including social support mechanisms and access to healthcare resources, which are often more limited for unmarried individuals (32, 33). Further research is needed to elucidate the pathways through which marital status influences the likelihood of ED involvement in cancer diagnosis and to develop targeted interventions to improve outcomes for unmarried patients (34, 35). We also found that unmarried patients were more likely to be diagnosed through the emergency department or diagnosed with advanced cancer than married patients. This finding has rarely been mentioned in the literature. Married people may receive social support from their spouses and have low cortisol levels, or perhaps high levels of natural killer cells, which may slow tumor progression. Spouses may also encourage patients to undergo early testing and treatment (36).

Personal medical history or family history of cancer may also affect cancer stage at the time of diagnosis (7, 24, 25). Patients with a history of hypertension or hyperlipidemia are more likely to be diagnosed with early-stage liver cancer (25) and patients with a history of polyp are more likely to be diagnosed with early-stage colorectal cancer (24). According to the literature, patients with more comorbidities are more likely to be diagnosed with tumors through the emergency department (37). Similar findings were noted in the present study: the proportion of patients newly diagnosed with advanced cancer was high (57.7%) for patients with a comorbidity severity score of 3 or more, followed by patients with a score of 2 (53.7%). The proportion of advanced cancers diagnosed was also high for patients with diabetes mellitus, stroke, hypertension, or chronic mental illness.

Prostate cancer had a significantly lower mortality risk than breast cancer; however, the risk was highest for lung cancer. Compared with other cancers, the probability of having advanced lung cancer at diagnosis is high (28). The same finding was noted in the present study. Compared with other cancer types, patients with lung cancer had an increased chance of being diagnosed with advanced cancer. Factors that lead to a delayed diagnosis of lung cancer should be prioritized when formulating policies and public health measures. Reasons for lung cancer being diagnosed at an advanced stage often include a combination of factors such as ignorance of initial symptoms and delays in seeking medical care, having diagnostic biopsies, and even getting referrals. All these factors could lead to having advanced cancer at diagnosis (38).

The current study showed that, regardless of cancer type, patients diagnosed with cancer through emergency departments had a significantly higher risk of death (2.24 times higher). A recent retrospective study found that, compared with patients diagnosed with cancer in non-emergency settings, patients diagnosed through emergency departments were at higher risk of being diagnosed with advanced cancer (relative risk, RR, 1.30; 95% CI, 1.39–1.58), and their survival rate was lower (RR, 0.61; 95% CI, 0.49–0.75) (37). In New Zealand, it was discovered that being diagnosed with cancer through emergency departments led to significantly lower survival rates among all ethnic groups (adj. OR 2.40, 95% CI 2.10–2.74) (39). The same condition could be found in Denmark, where the mortality rate within a year varied from 1.4% among patients who underwent screening to 53.0% among those who received a diagnosis following an unscheduled hospital admission. Individuals with an unscheduled admission had a higher likelihood of dying in the first year following diagnosis [OR = 3.38 (95% CI: 3.24–3.52)] compared to patients who were diagnosed through the cancer patient pathway from primary care (40). Similarly, the current study showed that approximately 76.4% of patients diagnosed with cancer via emergency departments had advanced cancer, and the risk of death was significantly high (HR, 1.46; 95% CI, 1.44–1.48). Another US study shows visiting emergency departments (ED) six months before a cancer diagnosis results in a higher mortality risk (OR = 1.73, 95% CI 1.38–2.18). Having Medicaid insurance leads to a higher rate (OR = 4.16, 95% CI 2.45–7.07). Over a third of cancer patients visit EDs before diagnosis (41). Also, a study illustrated that emergency presenters also had a greater risk of 12-month mortality than non-emergency presenters. Furthermore, a 10% increase in emergency presentations was associated with a decrease in one-year net survival of between 2.5% and 7% (20).

The survival curves of patients diagnosed through non-emergency and emergency departments revealed that the 5-year survival rate of patients diagnosed through the emergency department was lower than that of patients diagnosed through non-emergency departments. In terms of cancer types, compared with breast cancer, the mortality risk of prostate cancer was significantly lower, while those of oral, colorectal, and lung cancers were all significantly higher (P < 0.05). This finding was consistent with those of previous studies that found that patients diagnosed through the emergency department tended to have advanced disease and were at high risk of death (37, 42). Therefore, the emergency department plays a crucial role in the initial diagnosis of cancer and must be regarded as an important part of cancer care (43).

The current research’s findings, which are consistent with those of McPhail et al., show that patients over 75 years old, those with low incomes and education levels, those living in rural areas, and those with more comorbidities are more likely to receive a cancer diagnosis from the emergency department (20). For such patients, the initial diagnosis was often at advanced cancer stage. Therefore, patients over 75 years old, with a low education level, with low income, living in rural areas, or having more comorbidities may be classified as being at high risk for delayed cancer diagnosis. As Elliss-Brookes et al. emphasized, connecting patients with cancer with oncology care networks is a complex process, even for communities with complex cancer care systems (44). Another study found that certain sociodemographic and clinical factors, such as older age, non-Hispanic Black and Native Hawaiian/Other Pacific Islander race being unmarried, recent diagnosis year, later-stage disease, comorbidities, and poverty, were associated with an increased likelihood of ED involvement in cancer diagnosis (19). Cancer diagnoses often occur as emergencies across the globe, particularly among the elderly and those with advanced cancer, leading to negative impacts on survival rates. Enhancing cancer control on a global scale requires effective monitoring of emergencies, determining behavioral and healthcare factors, and prioritizing suitable interventions (20).

Patients in the abovementioned high-risk groups may not be able to attend cancer screenings and may ignore early symptoms, or they may not be able to get timely check-ups as they are busy earning a living. Hence, they are only connected to the medical care system through the emergency department. Therefore, it follows that these patients are mostly diagnosed with cancer through the emergency department, and they may not even undergo complete treatment. Recent literature suggests the need for a standardized referral process from emergency departments to cancer care systems, which may strengthen the connection between oncology and emergency teams and establish a multi-specialty medical cooperation system to improve the quality and efficiency of care (45). In the United States of America, the Comprehensive Oncologic Emergencies Research Network—established with support from the National Cancer Institute—promotes collaboration between the oncology and emergency medical departments to expand knowledge on the treatment of cancer in emergency medicine settings (46). On the other hand, there is a literature that has developed a digital quality measure (DQM) to help identify missed opportunities for diagnosis (MODs) and mortality due to diagnostic errors, such as overlooked test results, missed referrals, or miscommunication. Many patients with EP experience MODs, indicating that EP is linked to increased mortality and is responsible for preventable diagnostic delays. Nationwide implementation could result in strategies developed to address preventable cancer diagnostic delays (47).

### Study limitations

To the best of our knowledge, this is the first study conducted in Taiwan to compare the five major cancers diagnosed through outpatient clinics and emergency departments. The advantages of our study include that it was population-based and conducted over an extended period of time, and that it had a large sample size. However, certain limitations must be acknowledged.

Since the National Health Insurance Research Database was used to obtain data for analysis in the current study, only submitted data were presented, and we do not know whether patients underwent self-paid health examinations. Such patients were eventually diagnosed through outpatient clinics; however, it may have affected the proportion of diagnoses made through the emergency department. Furthermore, patients may have received interim treatment in an emergency department but were subsequently referred to an outpatient clinic for further confirmatory testing; this procedure may have resulted in an underestimation of the number of cancer cases diagnosed through emergency departments and an overestimation of the number of cancer cases diagnosed through outpatient clinics. Similar studies conducted in the United States of America determined that only 55% of the population was enrolled in the government’s medical insurance programs. The literature indicates that enrollment in a medical insurance plan has a significant impact on the cancer stage at diagnosis. People without medical insurance are more likely to be diagnosed with advanced cancer (48, 49). National Health Insurance in Taiwan has achieved full coverage, with an enrollment rate of 99.7% (50). For this reason, the findings of the current study cannot be extrapolated to other countries.

### Conclusions

The treatment outcome of patients with cancer is closely related to the cancer stage determined at the initial diagnosis. In the present study, patients with one of the top five cancers who were diagnosed through an emergency department often had a more advanced stage than those diagnosed through an outpatient clinic. Since the National Health Insurance Program has been implemented in Taiwan, almost 99.7% of people have been covered by health insurance. However, some people are still diagnosed with advanced cancer. Therefore, promoting regular health check-ups is important to reduce the risk of advanced cancer developing and only being identified at diagnosis. In addition, despite screening programs being implemented for four cancers, the proportion of cancer diagnoses made through the emergency department remains high. The government should consider further strengthening cancer screening for high-risk groups and people with low socioeconomic status to detect and treat cancer early. Patients diagnosed with one of the five major cancers through the emergency department mostly have advanced cancer and are at high risk of death. The trend of the proportion of cancer diagnoses through the emergency department may be used as an indicator for the effectiveness of cancer screening programs. Methods for strengthening the integration of the emergency department and cancer teams to reduce patients’ mortality risks must also be included as an important topic in cancer care.

## Data Availability

The original contributions presented in the study are included in the article/**Supplementary Material**. Further inquiries can be directed to the corresponding author.
