# Supplementary material for: Proportion trends, cancer stage, and survival of patients with cancer diagnosed through emergency and nonemergency departments: a nationwide cohort study
Source: Front Oncol. 2024 Aug 26;14:1399326. doi: 10.3389/fonc.2024.1399326 (PMC11381288; doi:10.3389/fonc.2024.1399326)
Supplement: Supplementary file 1 [file DataSheet1.pdf]

Supplementary Table A. Cancer diagnosis codes used in this study

Cancer Type	ICD-9	ICD-10
Breast Cancer	174.xx, 175.xx	C50.xx
Colorectal Cancer	153.xx, 154.xx	C18.xx, C19.xx, C20.xx, C21.xx
Lung Cancer	162.xx	C33.xx, C34.xx
Prostate Cancer	185.xx	C61.xx
Oral Cancer	140.xx, 141.xx, 142.xx, 143.xx, 144.xx, 145.xx, 146.xx, 147.xx, 148.xx, 149.xx	C00.xx, C01.xx, C02.xx, C03.xx, C04.xx, C05.xx, C06.xx, C07.xx, C08.xx, C09.xx, C10.xx, C11.xx, C12.xx, C13.xx, C14.xx
